# Comparison of diagnostic efficacy between ^99m^Tc-methylene diphosphate SPECT/CT and MRI for bone and joint infections: a multicenter retrospective analysis

**DOI:** 10.3389/fendo.2024.1359655

**Published:** 2024-02-29

**Authors:** Hao Gao, Guoquan Li, Congxiao Fu, Jun Ren, Fei Kang, Wen Luo, Qian Yin, Cheng Zhou, Bo Li, Shuaikun Lu, Hu Wang, Yong Zhang, Yunfei Zhang

**Affiliations:** ^1^Department of Orthopaedics, Second Affiliated Hospital, Air Force Medical University, Xi’an, Shaanxi, China; ^2^Department of Nuclear Medicine, First Affiliated Hospital, Air Force Medical University, Xi’an, Shaanxi, China; ^3^Department of Ultrasound, First Affiliated Hospital, Air Force Medical University, Xi’an, Shaanxi, China; ^4^Department of Radiology, Second Affiliated Hospital, Air Force Medical University, Xi’an, Shaanxi, China

**Keywords:** ^99m^Tc-MDP SPECT/CT, MRI, diagnostic efficacy, bone and joint infections, osteomyelitis

## Abstract

**Objective:**

There is currently no non-invasive examination that can fully determine the diagnosis of osteomyelitis. SPECT/CT tomographic fusion imaging can provide both local metabolic activity and anatomical information to determine the condition and location. This study evaluates the diagnostic efficacy of ^99m^Tc-MDP SPECT/CT in bone infections, compared to MRI.

**Methods:**

In this multicenter retrospective study, 363 patients with suspected bone and joint infections or osteomyelitis were included. Participants underwent ^99m^Tc-MDP SPECT/CT and/or MRI examinations, supplemented by pathogenic bacterial cultures and histopathological analysis.

**Results:**

Only SPECT/CT was tested in 169 patients, and only MRI was used in 116. 78 people have implemented both inspections and have detailed information. The diagnostic sensitivity and specificity of SPECT/CT for infection were 96% and 92% respectively, with an accuracy of 96%. For MRI, these figures were 88%, 84%, and 87% respectively.

**Conclusion:**

This represents the largest global study to date evaluating osteomyelitis and bone infection diagnosis using ^99m^Tc-MDP SPECT/CT tomographic fusion imaging. The findings indicate that ^99m^Tc-MDP SPECT/CT fusion imaging offers superior diagnostic accuracy compared to MRI. This is particularly evident in cases involving metallic implants and chronic infections. ^99m^Tc-MDP SPECT/CT fusion imaging emerges as a highly suitable non-invasive diagnostic modality, facilitating enhanced clinical follow-up and treatment.

## Introduction

Osteomyelitis and bone infections present significant challenges in contemporary orthopedic practice. In Europe, there has been a 10.44% increase in osteomyelitis incidence over recent years (1). In Asia, the rate ranges from 7.8 to 9.1 cases per 100,000 people annually (2). Patients may experience local discomfort, lack of mobility, severe complications, and substantial financial burden.

At present, there is no completely clear diagnosis to identify bone infection (3). Surgeons often depend on a comprehensive assessment encompassing clinical symptoms, laboratory investigations, bacterial cultures, and imaging findings, yet each of these methods has inherent limitations. The development of an early and precise diagnostic tool is of paramount importance.

SPECT/CT is capable of identifying musculoskeletal infections. It is particularly suitable for patients with multiple site infections and metallic implants (4). ^99m^Tc-MDP (^99m^Tc labeled methylene diphosphate) bone scintigraphy reveals radioactive uptake in areas of abnormal blood flow, altered bone salt metabolism, and osteogenesis associated with local bone lesions (5). PET also yields metabolic activity data, but it is not a specific bone imaging agent and is not suitable for assessing bone metabolism. Most notably, SPECT/CT stands out as a more cost-effective option (6). Therefore, SPECT/CT seems particularly valuable for delineating lesions and their associated inflammatory or infectious activities.

In recent years, there has been a surge in research exploring the role of nuclear medicine in osteomyelitis. However, these studies often suffer from small sample sizes and there is a lack of comparative studies with routine examination and corresponding subgroups. Especially after the widespread use of SPECT/CT fusion imaging technology. Consequently, we utilized the advantage of our existing large sample size to study the diagnostic efficacy of ^99m^Tc-MDP SPECT/CT for bone infection diseases and compared it with conventional MRI methods. Additionally, we examined the influence of variables such as the presence of metallic implants.

## Patients and methods

**Figure 1:**
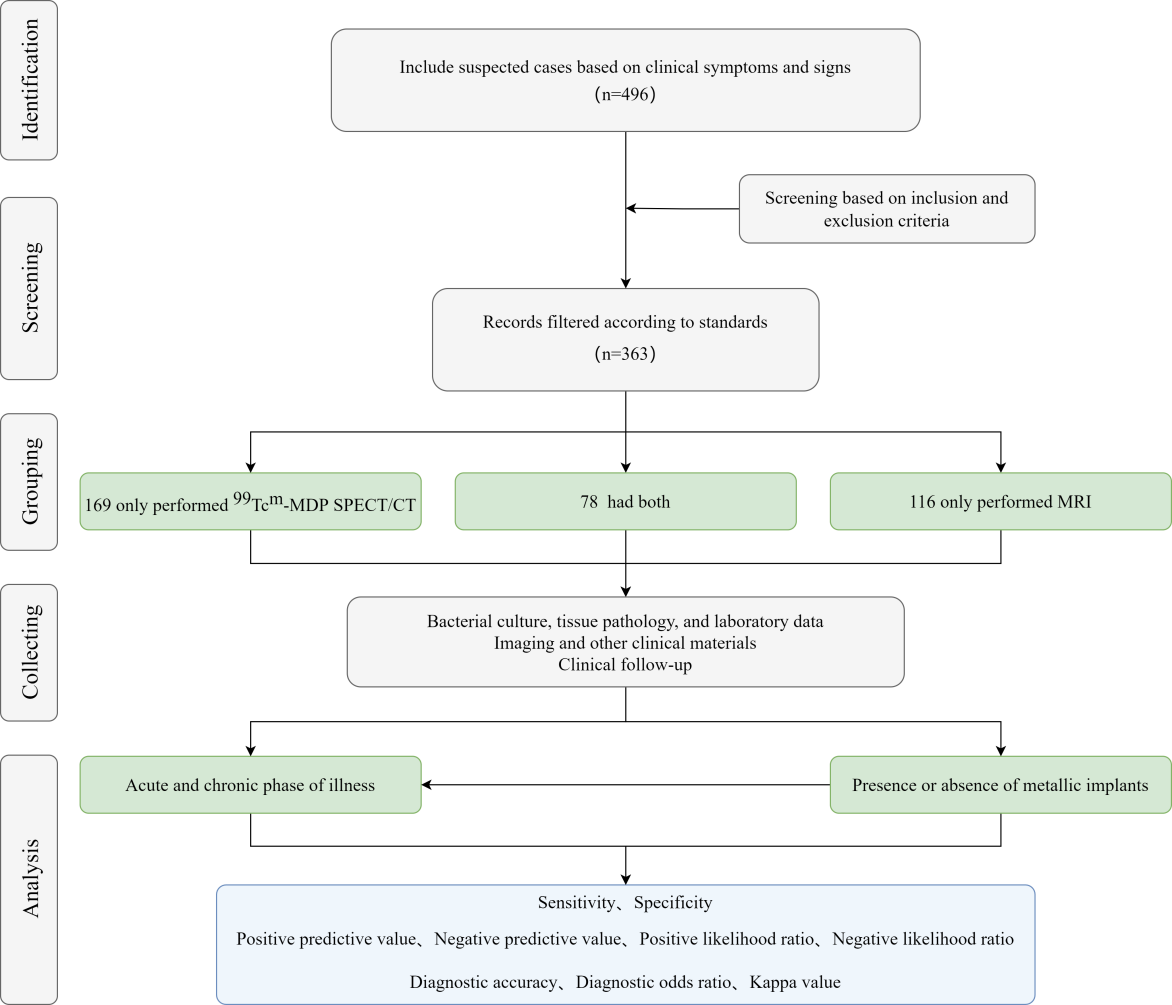
Diagnostic analysis flowchart for 363 patients.

This study is a multicenter retrospective study. Patients suspected of bone and joint infections or osteomyelitis admitted to the orthopedics department of two affiliated hospitals of the Medical University from January 2017 to June 2023 were included. Doctors suspect that the patient’s osteomyelitis or bone infection is due to clinical signs and abnormal laboratory test results (3). They underwent ^99m^Tc-MDP SPECT/CT and/or MRI examination, along with additional diagnostic procedures (**Figure 1**). The study protocol was approved by the Medical University’s Ethics Committee (K202309-12). Exclusion criteria included:

I: Patients with missing clinical data and incomplete medical records;
II: Patients who refuse to participate in the study;
III: Patients with other bone destruction diseases;
IV: Patients with specific bone infections such as tuberculosis.

Diagnosis of osteomyelitis or bone infection is based on preoperative or intraoperative microorganism cultivation and tissue pathology with clear evidence. Microbial cultures are conducted in the presence of bacteria or based on inflammatory signs in tissue samples (7). The determination of whether a patient carries a metal implant is based on visual imaging data. The determination of the acute and chronic stages of a patient’s onset is based on the onset time, with the first 8 weeks being the acute stage and over 8 weeks being the chronic stage (8).

### Nuclear medicine imaging

Adults received 20 to 25mCi of ^99m^Tc MDP intravenously. The dose for children was calculated as 250μCi/Kg. After 3-4 hours, the patient is placed in a supine position and a full body anteroposterior bone imaging is taken. SPECT/CT tomography is performed by collecting SPECT and CT images centered around the lesion and performing simultaneous fusion. The imaging instrument is SIEMENS SYMBIA T2 True Point SPECT/CT, and the SPECT/CT fusion image is processed using Syngo software.

### Magnetic resonance imaging

Using SIEMENS AG, German MAGNETOM Verio 1.5T for imaging, multiple sequences were executed. Routine cross-sectional and coronal T_1_WI and T_2_WI scans, with some additional sagittal scans. The area of interest was aligned with the coil center for optimal imaging. Routine coronal and cross-sectional T_1_WI and T_2_ STIR images of the hip and shoulder joints should be taken, and attention should be paid to symmetry on both sides during imaging. If necessary, sagittal scans should be added. Take routine T_1_WI and T_2_ STIR coronal and cross-sectional imaging of the knee joint, upper arm, and lower leg, and add sagittal scanning if necessary.

### Image analysis

Image analysis of SPECT/CT and MRI was performed independently by two experienced nuclear medicine physicians and two radiologists in a blinded manner. In case of inconsistent diagnosis, the results of comprehensive discussion shall prevail.

### Statistical analysis

The sensitivity, specificity, predictive value, likelihood ratio, diagnostic ratio, diagnostic accuracy, and Kappa value of SPECT/CT, MRI, and the combination of the two were calculated according to the previously mentioned reference standards. The statistical analysis software was SPSS version 26.0 (SPSS, Chicago, IL, USA), and the measurements were expressed as 95% confidence intervals, and statistically analyzed using the paired chi-square test. p<0.05 was considered statistically significant.

## Results

### Patient’s characteristics

A total of 169 suspected patients who only underwent ^99m^Tc-MDP SPECT/CT and 116 suspected patients who only underwent MRI are included. In addition, 78 patients had both examinations, providing comprehensive data (**Table 1**). The majority of cases were male (77.3%, 72.2%, and 73.1% in each group, respectively). There is no significant difference among the three groups. The age distribution was also relatively uniform, which was 43.30, 41.04, and 42.53 years old, respectively, and there is no difference among the three groups. There were 101 patients with implants in SPECT/CT, 19 patients with implants in MRI, and 12 patients with implants in both imaging modalities, and there is no significant difference among the three modalities. Diagnosis in all patients was confirmed through at least one of the two methods: clinical pathogen testing or histopathological examination. The proportion of patients who underwent pathogenic bacteria detection or pathological detection was the highest (96.7%), followed by SPECT/CT (92.0%) and MRI (90.0%). For patients diagnosed with osteomyelitis, the disease types were also analyzed. Hematogenous osteomyelitis accounted for a high proportion of MRI, with 101 cases. More patients with SPECT/CT had a history of trauma (148 cases). The number of cases of hematogenous osteomyelitis and traumatic osteomyelitis were 29 and 39, respectively.

**Table 1:** Main demographic characteristics of 363 patients.

	^99m^Tc-MDP SPECT/CT	MRI	MRI+^99m^Tc-MDP SPECT/CT
Total	247	194	78
Sex ratio (F/M)	56/191	54/140	21/57
Age			
Mean	43.3 ± 16.5	41.04 ± 20.39	42.53 ± 17.56
Range	3-77	2-85	4-75
Metal implants			
Yes	101	19	12
No	146	175	66
Final diagnosis method (Positive/Total)			
Microbiological test	84/90	62/69	21/26
Pathological examination	59/70	53/65	17/22
Microbiological test and pathological examination	80/87	54/60	29/30
Anatomical site (Number and percentage of items)			
Tibia and fibula	115 (46.6%)	65 (33.5%)	21 (26.9%)
Femur	89 (36.0%)	76 (39.2%)	34 (43.6%)
Ulnar and radius	6 (2.4%)	2 (1.0%)	0 (0%)
Humerus	6 (2.4%)	6 (3.1%)	1 (1.3%)
Others (Except vertebrae)	31 (12.6%)	45 (23.2%)	22 (28.2%)
Classification (Osteomyelitis diagnosed)	N=223	N=168	N=71
Hematogenous osteomyelitis	75	101	29
Traumatic osteomyelitis	148	67	39

### Comparison of diagnostic efficacy between ^99m^Tc-MDP SPECT/CT and MRI

In this study, a total of 247 people underwent ^99m^Tc-MDP SPECT/CT testing. This included 101 cases in the endoprosthesis group and 146 cases without endoprostheses. Correspondingly, in MRI, it is 19 and 175, respectively (**Table 2**). Except for MRI containing metallic implants, the P-values of statistical tests for all other categories were less than 0.01, indicating significant differences. In reality, only 19 patients with local defects containing implants underwent MRI imaging, a sample size too small for reliable statistical analysis.

**Table 2:** Diagnostic efficacy of ^99m^Tc-MDP SPECT/CT and MRI individually.

	^99m^Tc-MDP SPECT/CT			MRI		
	Metal implants (N=101)	No metal implants (N=146)	Total (N=247)	Metal implants (N=19)	No metal implants (N=175)	Total (N=194)
TP	87	128	215	16	132	148
FP	1	1	2	0	4	4
TN	8	14	22	1	20	21
FN	5	3	8	2	19	21
Sen	0.95 (0.87-0.98)	0.98 (0.93-0.99)	0.96 (0.93-0.98)	0.89 (0.64-0.98)	0.87 (0.81-0.92)	0.88 (0.81-0.92)
Spe	0.89 (0.51-0.99)	0.93 (0.66-1.00)	0.92 (0.72-0.99)	1.00 (0.05-1.00)	0.83 (0.62-0.95)	0.84 (0.63-0.95)
PPV	0.99 (0.93-1.00)	0.99 (0.95-1.00)	0.99 (0.96-1.00)	1.00 (0.76-1.00)	0.97 (0.92-0.99)	0.97 (0.93-0.99)
NPV	0.62 (0.32-0.85)	0.82 (0.56-0.95)	0.73 (0.54-0.87)	0.33 (0.02-0.87)	0.51 (0.35-0.67)	0.50 (0.34-0.66)
Acc	0.94 (0.89-0.99)	0.97 (0.94-1.00)	0.96 (0.93-0.98)	0.89 (0.76-1.00)	0.87 (0.82-0.92)	0.87 (0.82-0.92)
LR^+^	8.51 (1.34-54.04)	14.66 (2.21-97.37)	11.57 (3.07-43.62)	—	5.25 (2.14-12.86)	5.47 (2.23-13.46)
LR^-^	0.06 (0.02-0.15)	0.02 (0.01-0.08)	0.04 (0.02-0.08)	0.11 (0.03-0.41)	0.15 (0.10-0.23)	0.15 (0.10-0.22)
DOR	139.20 (14.44-1341.85)	597.33 (58.15-6136.26)	295.625 (59.07-1479.59)	—	34.74 (10.71-112.63)	37.00 (11.57-118.36)
Kappa	0.695 (P<0.01)	0.860 (P<0.01)	0.792 (P<0.01)	0.457 (P<0.05)	0.560 (P<0.01)	0.555 (P<0.01)

TP, True positive; FP, False positive; TN, True negative; FN, False negative; Sen, Sensitivity; Spe, Specificity; PPV, Positive predictive value; NPV, Negative predictive value; Acc, Diagnostic accuracy; LR^+^, Positive likelihood ratio; LR^-^, Negative likelihood ratio; DOR, Diagnostic odds ratio.

“—”, Unable to calculate.

Compared to the high sensitivity and specificity of SPECT/CT, MRI results were only 88% and 84%. Additionally, the diagnostic accuracy, negative predictive value (NPV), and likelihood ratio for MRI were also lower than those for SPECT/CT. Notably, MRI generally underperformed in consistency testing compared to the gold standard. In contrast, SPECT/CT showed excellent concordance with resultant values >0.75.

SPECT/CT’s superior diagnostic performance was consistent irrespective of the presence or absence of metal endoprostheses. Specifically, in the presence of metallic implants, the diagnostic accuracy, positive likelihood ratio, negative likelihood ratio, and Kappa value were 94%, 8.51, 0.06, and 0.695, respectively, although the sensitivity and specificity were similar to those of MRI. In cases without endoprostheses, the diagnostic efficacy of SPECT/CT was even more pronounced. This indicates that SPECT/CT has excellent and reliable diagnostic ability for bone and joint infections, especially in the presence of metal implants.

Additionally, our team performed an analysis of the diagnostic efficacy in both acute and chronic onset scenarios (**Table 3**). The results showed that there was no significant difference in diagnostic sensitivity and specificity between SPECT/CT and MRI, and the former remained superior. In the acute phase, the Kappa value of SPECT/CT and the gold standard was 0.897, which is better than 0.601 and 0.530 of MRI in the acute phase and chronic phase, respectively. In addition, in terms of predicted values and likelihood ratios, there are no significant differences between the two tests in both subgroups. It is particularly pointed out that in the chronic stage of the disease, with the presence of metal implants, the positive likelihood ratio of SPECT/CT is as high as 11.77, indicating a high diagnostic value.

**Table 3:** Diagnostic efficacy of ^99m^Tc-MDP SPECT/CT and MRI individually in the chronic phase of illness.

^99m^Tc-MDP SPECT/CT				MRI		
The acute phase of illness.						
Metal implants (N=15)		No metal implants (N=31)	Total (N=46)	Metal implants (N=5)	No metal implants (N=63)	Total (N=68)
TP	13	27	40	5	47	52
FP	0	0	0	0	1	1
TN	2	3	5	0	8	8
FN	0	1	1	0	7	7
Sen	1.00(0.72-1.00)	0.96(0.80-0.99)	0.98(0.86-0.99)	1.00(0.46-1.00)	0.87(0.74-0.93)	0.88(0.76-0.95)
Spe	1.00(0.20-1.00)	1.00(0.31-1.00)	1.00(0.46-1.00)	—	0.89(0.51-0.99)	0.89(0.51-0.99)
PPV	1.00(0.72-1.00)	1.00(0.85-1.00)	1.00(0.89-1.00)	1.00(0.46-1.00)	0.98(0.88-0.99)	0.98(0.89-0.99)
NPV	1.00(0.20-1.00)	0.75(0.22-0.99)	0.83(0.36-0.99)	—	0.53(0.27-0.78)	0.53(0.27-0.78)
Acc	1.00(1.00-1.00)	0.97(0.91-1.00)	0.98(0.94-1.00)	1.00(1.00-1.00)	0.87(0.79-0.96)	0.88(0.81-0.96)
LR^+^	—	—	—	—	7.83(1.23-49.86)	7.93(1.25-50.46)
LR^-^	—	0.04(0.005-0.24)	0.02(0.004-0.17)	—	0.15(0.07-0.30)	0.13(0.06-0.27)
DOR	—	—	—	—	53.71(5.80-497.24)	59.43(6.43-549.1618)
Kappa	1.000(P<0.01)	0.839(P<0.01)	0.897(P<0.01)	—	0.594(P<0.01)	0.601(P<0.01)
The chronic phase of illness.						
	(N=86)	(N=115)	(N=201)	(N=14)	(N=112)	(N=126)
TP	74	101	175	11	85	96
FP	1	1	2	0	3	3
TN	6	11	17	1	12	13
FN	5	2	7	2	12	14
Sen	0.94(0.85-0.98)	0.98(0.92-1.00)	0.96(0.92-0.98)	0.85(0.54-0.97)	0.88(0.79-0.93)	0.87(0.79-0.93)
Spe	0.86(0.42-0.99)	0.92(0.60-1.00)	0.89(0.65-0.98)	1.00(0.05-1.00)	0.80(0.51-0.95)	0.81(0.54-0.95)
PPV	0.99(0.92-1.00)	0.99(0.94-1.00)	0.99(0.96-1.00)	1.00(0.68-1.00)	0.97(0.90-0.99)	0.97(0.91-0.99)
NPV	0.55(0.25-0.82)	0.85(0.54-0.97)	0.71(0.49-0.87)	0.33(0.02-0.87)	0.50(0.30-0.70)	0.48(0.29-0.68)
Acc	0.93(0.88-0.98)	0.97(0.94-1.00)	0.96(0.93-0.98)	0.86(0.67-1.00)	0.87(0.80-0.93)	0.87(0.81-0.92)
LR^+^	6.56(1.07-40.29)	11.77(1.80-76.86)	9.13(2.46-33.90)	—	4.38(1.6-12.10)	4.65(1.67-12.94)
LR^-^	0.07(0.03-0.18)	0.02(0.005-0.08)	0.04(0.02-0.09)	0.15(0.04-0.55)	0.15(0.10-0.27)	0.16(0.09-0.26)
DOR	88.80(8.88-888.04)	555.50(46.53-6632.40)	212.50(40.87-1104.82)	—	28.33(6.97-115.14)	29.71(7.51-117.515)
Kappa	0.630(P<0.01)	0.865(P<0.01)	0.766(P<0.01)	0.440(P<0.05)	0.539(P<0.01)	0.530(P<0.01)

TP, True positive; FP, False positive; TN, True negative; FN, False negative; Sen, Sensitivity; Spe, Specificity; PPV, Positive predictive value; NPV, Negative predictive value; Acc, Diagnostic accuracy; LR^+^, Positive likelihood ratio; LR^-^, Negative likelihood ratio; DOR, Diagnostic odds ratio.

“—”, Unable to calculate.

### Diagnostic efficacy of combined use of ^99m^Tc-MDP SPECT/CT and MRI

We also analyzed the diagnostic efficacy of the combination of MRI and SPECT/CT. A cohort of 78 patients who underwent both MRI and SPECT/CT during the same period and for whom complete data were available was analyzed. The relevant data were obtained by the calculation principle of the tandem combination test (**Table 4**). The specific implementation method of the series combination test is based on the positive MRI findings. If the SPECT/CT results are positive, the combined detection will yield a positive outcome; conversely, if the SPECT/CT results are negative, the combined detection will yield a negative outcome. The combined use resulted in overall specificity, sensitivity, and diagnostic accuracy superior to MRI alone and comparable to SPECT/CT alone (98%, 100%, and 98%, respectively). Similarly, we analyzed the case of the endophyte. Without implants, the diagnostic sensitivity, specificity, and accuracy of MRI and SPECT/CT were generally consistent and close to the parametric results of SPECT/CT alone and significantly better than MRI alone. It was evident in this group that the negative likelihood ratio of MRI alone was as high as 0.34 and the Kappa value as low as 0.242. The results of the comparison between the acute and chronic conditions are presented in **Table 5**, and the results are consistent with the above. When combined, it has a higher diagnostic ability than MRI in the acute and chronic phases of the disease and is better than the whole.

**Table 4:** Diagnostic efficacy of combined use of ^99m^Tc-MDP SPECT/CT and MRI.

	^99m^Tc-MDP SPECT/CT	MRI	MRI+^99m^Tc-MDP SPECT/CT		
	Total (N=78)	Total (N=78)	Metal implants (N=10)	No metal implants (N=46)	Total (N=56)
TP	70	54	10	43	53
FP	0	2	0	0	0
TN	7	5	0	2	2
FN	1	17	0	1	1
Sen	0.99 (0.91-1.00)	0.76 (0.64-0.85)	1.00 (0.66-1.00)	0.98 (0.86-1.00)	0.98 (0.89-0.99)
Spe	1.00 (0.56-1.00)	0.71 (0.30-0.95)	—	1.00 (0.20-1.00)	1.00 (0.20-1.00)
PPV	1.00 (0.94-1.00)	0.96 (0.87-0.99)	1.00 (0.66-1.00)	1.00 (0.90-1.00)	1.00 (0.92-1.00)
NPV	0.88 (0.47-0.99)	0.23 (0.09-0.46)	—	0.67 (0.13-0.98)	0.67 (0.13-0.98)
Acc	0.99 (0.96-1.00)	0.76 (0.66-0.85)	1.00 (1.00-1.00)	0.98 (0.94-1.00)	0.98 (0.95-1.00)
LR^+^	—	2.66 (0.82-8.65)	—	—	—
LR^-^	0.01 (0.00-0.10)	0.34 (0.20-0.57)	—	0.02 (0.00-0.16)	0.02 (0.00-0.13)
DOR	—	7.94 (1.41-44.71)	—	—	—
Kappa	0.926 (P<0.01)	0.242 (P<0.01)	—	0.789 (P<0.01)	0.791 (P<0.01)

TP, True positive; FP, False positive; TN, True negative; FN, False negative; Sen, Sensitivity; Spe, Specificity; PPV, Positive predictive value; NPV, Negative predictive value; Acc, Diagnostic accuracy; LR^+^, Positive likelihood ratio; LR^-^, Negative likelihood ratio; DOR, Diagnostic odds ratio.

“—”, Unable to calculate.

**Table 5:** Diagnostic efficacy of combined use of ^99m^Tc-MDP SPECT/CT and MRI in acute and chronic phases of illness.

	Patients in the acute phase of illness			Patients in the chronic phase of illness		
	Metal implants (N=2)	No metal implants (N=12)	Total (N=14)	Metal implants (N=8)	No metal implants (N=34)	Total (N=42)
TP	2	11	13	8	32	40
FP	0	0	0	0	0	0
TN	0	1	1	0	1	1
FN	0	0	0	0	1	1
Sen	1.00 (0.20-1.00)	1.00 (0.68-1.00)	1.00 (0.72-1.00)	1.00 (0.60-1.00)	0.97 (0.82-1.00)	0.98 (0.86-1.00)
Spe	—	1.00 (0.05-1.00)	1.00 (0.05-1.00)	—	1.00 (0.05-1.00)	1.00 (0.05-1.00)
PPV	1.00 (0.20-1.00)	1.00 (0.68-1.00)	1.00 (0.72-1.00)	1.00 (0.60-1.00)	1.00 (0.87-1.00)	1.00 (0.89-1.00)
NPV	—	1.00 (0.05-1.00)	1.00 (0.05-1.00)	—	0.50 (0.03-0.97)	0.50 (0.03-0.97)
Acc	1.00 (1.00-1.00)	1.00 (1.00-1.00)	1.00 (1.00-1.00)	1.00 (1.00-1.00)	0.97 (0.91-1.00)	0.97 (0.93-1.00)
LR^+^	—	—	—	—	—	—
LR^-^	—	—	—	—	0.03 (0.00-0.21)	0.02 (0.00-0.17)
DOR	—	—	—	—	—	—
Kappa	—	1.000 (P<0.01)	1.000 (P<0.01)	—	0.653 (P<0.01)	0.656 (P<0.01)

TP, True positive; FP, False positive; TN, True negative; FN, False negative; Sen, Sensitivity; Spe, Specificity; PPV, Positive predictive value; NPV, Negative predictive value; Acc, Diagnostic accuracy; LR^+^, Positive likelihood ratio; LR^-^, Negative likelihood ratio; DOR, Diagnostic odds ratio.

“—”, Unable to calculate.

## Discussion

The clinical symptoms of bone and joint infection are not typical and are easily confused with other diseases. The detection rate of conventional imaging modalities for early lesions is low. Microbial culture and tissue biopsy are time-consuming processes. These challenges make the diagnosis more difficult and also reflect the necessity of our research. As far as we know, this study is currently the largest sample size in the diagnostic efficacy of ^99m^Tc-MDP SPECT/CT for bone infections worldwide, and it is also one of the few studies to compare with MRI. More importantly, we also analyzed the impact of metal implants and acute and chronic disease factors, as well as whether combined use with MRI can improve diagnostic efficacy.

SPECT/CT technology can provide more accurate diagnostic results. This technology amalgamates the advantages of SPECT and CT, offering images that concurrently delineate metabolic activity and cross-sectional anatomical structure, allowing for a more accurate assessment of the nature, location, size, and degree of acute and chronic activity of bone-positive lesions (9). According to reports, SPECT/CT can increase the diagnostic specificity from 78% to 89% and maintain 100% high sensitivity compared to SPECT alone (10). Since the 1970s, ^99m^Tc labeled MDP has been widely used as an imaging agent in various orthopedic metabolic diseases in clinical practice (9, 11).

The results of this article indicate that the overall sensitivity, specificity, and diagnostic accuracy of ^99m^Tc-MDP SPECT/CT are significantly superior to MRI, and its diagnostic consistency with the gold standard is also as high as 0.792. This confirms that SPECT/CT has an extremely high diagnostic ability for bone infection compared to conventional imaging examinations. Overall, our conclusion is better than Paez’s diagnostic efficacy for individual items in postoperative vertebral infection, and also better than Lee’s conclusion. Especially in the presence of metallic implants, the diagnostic ability of SPECT/CT is particularly outstanding, with sensitivity and specificity of 95% and 89%, respectively, and consistency of 0.695, which is significantly higher than that of the MRI group (12, 13). However, the total sensitivity and specificity of ^99m^Tc labeled anti-granulocyte SPECT/CT examination on suspected osteomyelitis and implant infection patients by Plate were only 77.8% and 94.1%, respectively (14).

Previously, MRI was recognized for its high sensitivity in detecting early infection, including local edema, exudation, and soft tissue abnormalities (15). However, this also made it easy to misdiagnose, especially metal implants that could introduce artifacts that reduced diagnostic accuracy, making it difficult to differentiate from sterile inflammation (15–17). Therefore, relying solely on MRI to evaluate bone infection is limited, especially in patients with metal implants. Our data further indicates that the diagnostic accuracy of MRI in patients with endoprostheses is just 89%, with a consistency of only 0.457 when compared to the gold standard. This significantly trails behind the 96% accuracy and 0.766 consistency of ^99m^Tc-MDP SPECT/CT, aligning with earlier reports (18).

From the perspective of onset time, both ^99m^Tc-MDP SPECT/CT and MRI have good diagnostic capabilities in the early stages of the disease. Based on the patient’s symptoms and signs, the sensitivity and specificity of SPECT/CT diagnosis are as high as 98% and 100%, which are higher than 88% and 89% of MRI. The diagnostic consistency with the gold standard is also as high as 0.897. Compared to the high diagnostic ability in the acute phase, the disappearance of local symptoms in the chronic phase weakens, and the difficulty of imaging judgment increases. The consistency between the two imaging methods and the gold standard diagnosis has decreased, but SPECT/CT still maintains good results, with a Kappa value of up to 0.766. This is because in both acute and chronic stages, the blood flow and bone salt metabolism at the local infection site are higher than others, while edema and inflammatory exudation are not significant. In clinical practice, chronic osteomyelitis only manifests as cortical thickening on MRI. A single sign makes it difficult for radiologists to obtain accurate diagnosis. Therefore, we believe that compared to MRI, SPECT/CT can detect infection in the early stage and provide accurate diagnosis in the chronic stage, providing an accurate basis for the design of antibacterial treatment plans in the later stage. Especially in the presence of metallic implants, the diagnostic accuracy and consistency with the gold standard of SPECT/CT are superior to MRI in both acute and chronic infections.

In addition, the diversity of trauma mechanisms and the complexity of surgical repair in real life can make it difficult to determine the extent of bone infection. The complete debridement range of surgery is one of the important factors affecting postoperative recurrence (19). MRI cannot distinguish whether the high signal area is caused by edema or inflammation on imaging. Filippi et al. reported that ^99m^Tc labeled white blood cell SPECT/CT can accurately delineate the infection range (20); Kim et al. also reported that labeled white blood cell SPECT/CT imaging can accurately locate infectious lesions and delineate the scope of infection (21). Similarly, during the research process, We also found that in addition to its excellent diagnostic performance, SPECT/CT can accurately define the degree of infection by using its radiation count distribution isolines (ISO). This is because the intake of MDP changes with the degree of inflammatory stimulation, presenting a gradual state of central strength and peripheral weakness. We infer that this approach can provide a reliable basis for thorough debridement in clinical practice, thereby avoiding overtreatment and improving the cure rate of chronic hematogenous osteomyelitis.

**Figure 2:**
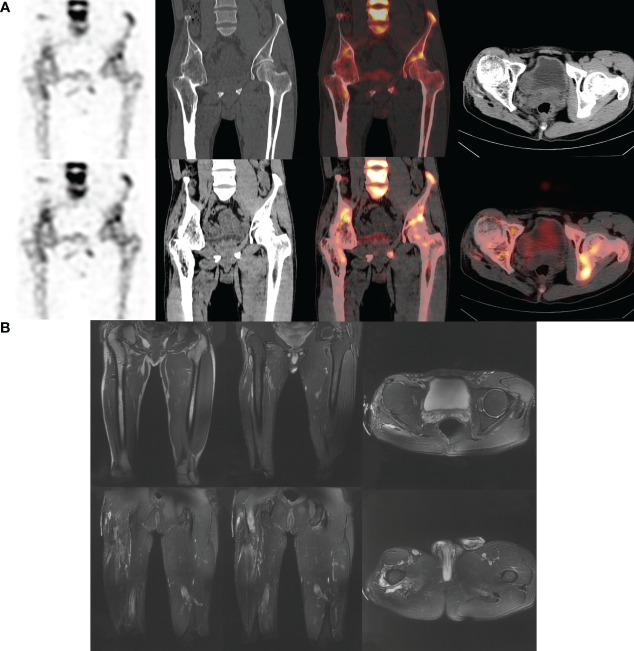
Male. 43y. There is no obvious cause of right lower limb pain, local swelling with pus discharge, and the pain worsens after activity. No fever. More than 10 years ago, the right femoral shaft fracture underwent reduction and internal fixation surgery and was removed. The physical examination was negative. WBC: 8.37×10^9^/L; LYM%: 16.7%; MONO%: 6.7%; NE%: 74.9%; ESR: 16.0 mm/hr; hs-CRP: 0.69 mg/L. Pathological examination showed infiltration of neutrophils, visible necrosis and abscess formation, and inflammatory changes. **(A)** SPECT/CT shows bone changes in the right proximal femur and right hip joint area with abnormal bone metabolism, which is consistent with chronic infectious disease. **(B)** MRI considers bony fusion of the right hip joint; Bone protrusions in the upper and middle segments of the right femur, considering ectopic ossification; Edematous changes in the right soft tissue.

**Figure 3:**
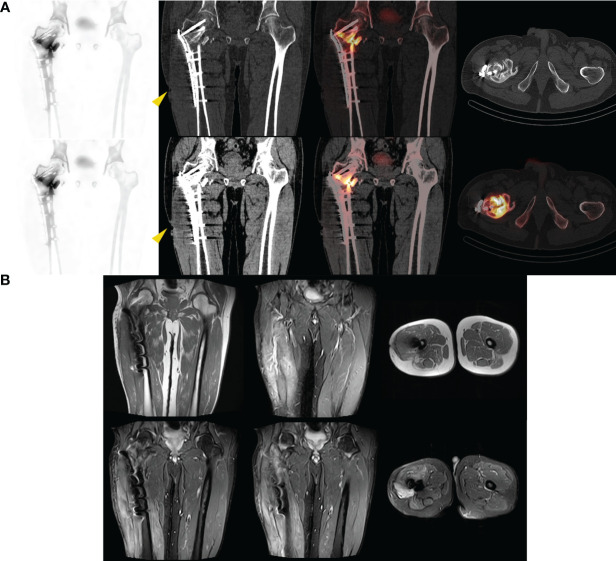
Male. 61y. After 9 months of open reduction and internal fixation for intertrochanteric fracture of the femur. Pain at the right thigh wound, with purulent discharge visible. A sinus formation can be seen in the middle section of the scar on the left thigh after surgery, with a small amount of secretion oozing out. WBC: 4.79×10^9^/L; LYM%: 32.6%; MONO%: 6.7%; NE%: 54.1%; hs-CRP: 2.11mg/L. Bacterial culture suggests Escherichia coli. **(A)** SPECT/CT suggests that after the right proximal femur surgery, there are bone changes in the surgical area accompanied by abnormal bone metabolism, as well as low-density soft tissue shadows and sinus formation (yellow arrow), suggesting an infectious lesion. **(B)** MRI considers changes after internal fixation surgery, resulting in swelling of the thigh soft tissue. Edema of subcutaneous soft tissue on the outer side of the right thigh.

Furthermore, the overall specificity and sensitivity of patients using a combination of two tests are superior to those using MRI alone, and their diagnostic ability is similar to that of ^99m^Tc-MDP SPECT/CT alone. After conducting a subgroup analysis of whether or not there were metallic implants, it was found that in the absence of metallic implants, patients who underwent both tests had a similar diagnostic ability to the overall population and were close to the parameter results of SPECT/CT alone, as well as significantly superior to MRI detection alone. It is worth noting that in the joint analysis, the results of using MRI alone with corresponding low values once again indicate that it cannot effectively distinguish between real infections and other inflammatory diseases. As shown in our case (**Figure 2**), the patient had no obvious cause of pain accompanied by local pus discharge. The MRI findings only showed local soft tissue edema, while SPECT/CT showed local bone changes and soft tissue infections. In another case (**Figure 3**), more than 9 months after the patient underwent open reduction and internal fixation surgery, the pain was found at the right thigh wound, with pus discharge and sinus formation visible. Obvious metal artifacts can be seen in MRI, and local observations can only show obvious edema, while SPECT/CT fusion tomography imaging can clearly show bone changes in the surgical area with abnormal bone metabolism, low-density shadows in soft tissue, and sinus formation, which is in line with infectious changes.

Through the analysis of a large sample size of data, our data can strongly demonstrate that ^99m^Tc-MDP SPECT/CT has great advantages in diagnosing infectious bone diseases and other related aspects compared to MRI. Less impacted by metal implants, ^99m^Tc-MDP SPECT/CT can accurately assess the nature and location of the condition and delineate the infection extent, aiding clinicians in treatment planning and enhancing cure rates. Our data conclusion supports a new diagnostic process, where patients with clinical symptoms pointing to infection and metal implants or in the chronic stage of the disease can implement SPECT/CT as soon as possible. This not only enables timely diagnosis but also reduces the patient’s excessive energy and economic consumption. However, it is undeniable that in clinical practice, MRI can display the adjacent structures around bones, such as the imaging characteristics and anatomical relationships of nerves and blood vessels. Therefore, we believe that adding MRI to the ^99m^Tc-MDP SPECT/CT diagnosis and subsequent treatment is necessary, and this combined approach is helpful for the diagnosis and treatment of patients.

This study also has certain limitations. Firstly, although we use previous experience to comprehensively assess the patient’s condition to include patients, we mainly rely on the judgment of clinical doctors, which may lead to bias. Nonetheless, the accuracy observed in our study aligns with the overall accuracy reported in existing literature. Furthermore, the exclusion of specific infections from our study suggests the possibility of alternative conclusions. Most importantly, this study is retrospective, and there may be various circumstances that affect the conclusion, such as the deviation of the completeness and homogeneity of data collection, and the difficulty in ensuring the consistency of doctors’ examination methods and diagnostic criteria. Further prospective studies should be conducted to verify the results.

## Conclusion

In summary, ^99m^Tc-MDP SPECT/CT tomographic fusion imaging is a highly suitable non-invasive diagnostic modality. Especially in the chronic stage of metal implant and infection, its diagnostic ability is more prominent than MRI. Early adoption after a comprehensive assessment of the situation may be more helpful for clinical follow-up treatment.

## Ethical approval statement

The institutional review board of the ethics committee at our institution approved this study (No. K202309-12).

## Data availability statement

The raw data supporting the conclusions of this article will be made available by the authors, without undue reservation.

## Ethics statement

The studies involving humans were approved by the Medical Ethics Committee of the Second Affiliated Hospital, Air Force Medical University. The studies were conducted in accordance with the local legislation and institutional requirements. Written informed consent for participation was not required from the participants or the participants’ legal guardians/next of kin in accordance with the national legislation and institutional requirements. Written informed consent was obtained from the individual(s) for the publication of any potentially identifiable images or data included in this article.

## Author contributions

HG: Data curation, Investigation, Writing – original draft, Writing – review & editing, Methodology, Project administration, Software, Validation, Visualization. GL: Methodology, Formal analysis, Investigation, Resources, Writing – original draft. CF: Data curation, Investigation, Validation, Writing – original draft, Writing – review & editing. JR: Data curation, Investigation, Writing – original draft, Validation. FK: Resources, Writing – review & editing. WL: Formal analysis, Resources, Writing – review & editing. QY: Formal analysis, Resources, Writing – review & editing. CZ: Formal analysis, Investigation, Writing – review & editing. BL: Formal analysis, Investigation, Writing – review & editing. SL: Validation, Writing – review & editing. HW: Investigation, Writing – review & editing. YFZ: Conceptualization, Methodology, Resources, Supervision, Writing – review & editing, Data curation, Funding acquisition, Project administration, Writing – original draft. YZ: Conceptualization, Methodology, Resources, Supervision, Writing – review & editing.
